# Supplementary material for: Light-responsive self-strained organic semiconductor for large flexible OFET sensing array
Source: Nat Commun. 2022 Aug 20;13:4912. doi: 10.1038/s41467-022-32647-9 (PMC9392737; doi:10.1038/s41467-022-32647-9)
Supplement: Supplementary file 1 — Supplementary Information [file 41467_2022_32647_MOESM1_ESM.pdf]

Supplementary Information

Light-responsive Self-Strained Organic Semiconductor for Large Flexible OFET Sensing Array

Li et al.

Supplementary Methods

Molecular synthesis. All reagents and chemicals were obtained from commercial sources and used without further purification unless otherwise noted. All reactions were performed under an inert atmosphere of argon in dry solvents using standard Schlenk techniques. ^{1}H and ^{13}C spectra were recorded on Bruker–400 MHz NMR ARX400. Chemical shifts of ^{1}H and ^{13}C NMR signals were quoted to tetramethylsilane (δ = 0.00 ppm) and $CDCl_3$ (δ = 77.00 ppm) as internal standards, respectively. Mass spectra were recorded on a Bruker APEX IV mass spectrometer. The synthetic route used to obtain linker AZO-BTBT-8 is outlined in Scheme S1. **Compound 1-7** were synthesized according to the literatures.[1,2]

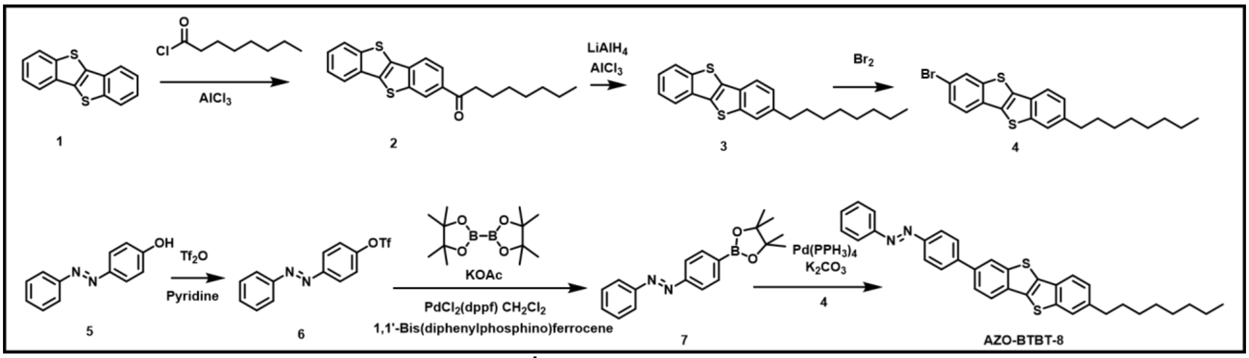

Supplementary Figure 1 | The synthesis route of AZO-BTBT-8

AZO-BTBT-8 was synthesized by a standard Suzuki reaction. **Compound 4** (200 mg, 0.41 mmol), **compound 7** (263 mg, 0.45 mmol), K_2CO_3 (120 mg, 0.87 mmol) and $Pd(PPh_3)_4$ (15 mg, 0.013 mmol) were added in a specially designed conical flask protected with nitrogen. 30 mL degassed solution (V_{THF}: V_{water} = 5:1) was added after the apparatus was sealed. The system was held at 80 °C and carefully stirring for 24 h. ^{1}H NMR ($CDCl_3$, 400 MHz, ppm): δ 8.16 (d, 2H, J = 8.2 Hz), 8.03 (d, 2H, J = 7.3 Hz), 7.97 (d, 1H, J = 7.8 Hz), 7.78 (m, 3H), 7.57 (m, 4H), 7.46 (m, 2H), 7.28 (s, 1H), 2.72 (t, 2H, J = 7.6 Hz), 1.66 (m, 2H), 1.28 (m, 10H), 0.91 (m, 3H). ^{13}C NMR ($CDCl_3$, 125 MHz, ppm): δ 152.78, 152.34, 142.91, 142.67, 142.59, 140.53, 136.25, 134.05, 131.89, 131.76, 131.15, 130.28, 130.24, 129.18, 125.91, 125.82, 124.61, 123.45, 123.12, 122.99, 122.81, 120.89, 36.10, 31.89, 31.61, 29.73, 29.48, 29.26, 22.68, 14.13. EI-HRMS: calcd. for $[M]^+$:532.2001. Found: 532.2008.

UV–Vis absorption. UV–Vis absorption measurements of solution (acetonitrile as solvent) and thin films (on quartz substrates) were determined with a Perkin–Elmer Lambda 950 UV/Vis spectrometer. The thin films were directly prepared by spin-coating (3k rpm, 120 s).

Scanning tunneling microscopy (STM). STM experiments were performed to obtain the molecular packing on highly oriented pyrolytic graphite (HOPG). A drop of the AZO-BTBT-8 solution (10^{-4} M in octylbenzene) was deposited onto a freshly cleaved surface of HOPG (quality ZYB, Digital Instruments). After evaporation of the solvent at 60 °C, a self-assembled monolayer was formed and used for STM measurements. The samples were observed by JPK AFM on STM module. The tunneling tips were prepared by mechanically cutting Pt/Ir wire (90/10). All the STM images were recorded in the constant-current mode.

Cyclic voltammetry (CV). CV measurements were conducted by an electrochemical analyser (Multi PalmSens4) in deionized water or acetonitrile containing 0.05 M tetrabutylammonium perchlorate as a supporting electrolyte. The solution concentration is $3.2*10^{-3}$ M. A glassy carbon electrodes was used as a working electrode and a platinum wire as a counter electrode; all potentials were recorded versus Ag/AgCl as a reference electrode. Before measurements, the solution was deoxygenated by nitrogen bubbling for 10 minutes. The energy levels of organic semiconductors were calculated by using the ferrocenemethanol value of –4.8 eV as the standard. The scan rate was 50 mV s^{-1}. The film samples were prepare by droping solution onto the glassy carbon electrodes overnight.

X–ray photoelectron spectroscopy (XPS): XPS data were obtained with an Axis Ultra Imaging X–ray Photoelectron Spectrometer from Kratos Analytical Ltd by using 300 W AlKα radiation. The base pressure was about 3×10^{-9} mbar. The binding energies were referenced to the C1s line at 284.8 eV from adventitious carbon.

Thermogravimetric analysis (TGA): TGA measurements were conducted on TA Instruments Q600 SDT thermal analysis system under N_2 at a heating rate of 10 °C min^{-1}.

Differential scanning calorimetry (DSC): DSC measurements were conducted by using a TA Instruments Q2000 differential scanning calorimeter under N_2.

Scanning electron microscope (SEM): Merlin Compact ZEISS Field-emission SEM was used with a scan voltage of 1.0 kV.

The optical microscopy (OM) and **the polarized optical microscopy (POM)** with heat stage were obtained on silicon substrates by using Nikon Eclipse LV100 POL, Japan in reflection mode on a home-made heat stage.

Calculation method for the percent conversion of AZO-BTBT-8 molecules:

The fitting and calculation can be determined from UV-Vis spectra, current plots and fitting curves from *in-situ* conductive AFM.

Photochromism is defined as a reversible change in a chemical species between two forms.

$$\text{AZO-BTBT-8-}\textit{trans}\ (A) \underset{k_{\text{Vis}}}{\overset{k_{\text{UV}}}{\rightleftharpoons}} \text{AZO-BTBT-8-}\textit{cis}\ (B)$$

To calculate the percent conversion (x_e) from AZO-BTBT-8-*trans* (A) to AZO-BTBT-8-*cis* (B), a semiempirical approach is used, assuming that a pseudo-first-order process with linear intensity dependence adequately describes the present photochromic system. The overall change in concentration of AZO-BTBT-8-*cis* (B) with time can be simply given by the following rate equation:

$$dB/dt = k_{UV}(A_0 - B) \tag{1}$$

Integration of the rate equation yields:

$$\ln\frac{A_0}{A_0 - B} = k_{UV}t \tag{2}$$

$$\text{or} \quad B = A_0 - A_0\exp(-k_{UV}t) \tag{3}$$

where A and B are the concentrations of AZO-BTBT-8-*trans* and AZO-BTBT-8-*cis*, k_{UV} is the photochemical rate constant, A_0 is the total concentration of all the AZO-BTBT-8 molecules. In this case the absorbance maximum was used as analyzing wavelength of plot peaks accordingly in Supplementary Fig. 9a.

$$[Abs] = a - b\exp(-k_{UV}t) \tag{4}$$

Where a, b are fitting constants. At the photostationary state, the percent conversion (x_e) from AZO-BTBT-8-*trans* to AZO-BTBT-8-*cis* can be calculated through the following relationship:

$$x_e = \frac{A}{A_0} = [\exp(k_{UV}t)]^{-1} \times 100\% \tag{5}$$

Half-life time (which means the time of half transformation of the active AZO-BTBT-8-*trans* to AZO-BTBT-8-*cis*):

$$t_{1/2} = \frac{ln2}{k_{UV}} \tag{6}$$

The bending method and stain estimation:

To evaluate the bending stability of the OFETs, the PET substrate was bent from flat to curved along an axis running exactly through the transistors by a home-made setup (Supplementary Fig. 19). The strain *S* induced in a particular layer having a thickness t_L located on the surface of a substrate having a thickness t_S by bending the substrate into a radius *R* is given by the following equation:

$$S = \frac{(t_L+t_S)(1+2\eta+\chi\eta^2)}{2R(1+\eta)(1+\chi\eta)} \tag{7}$$

where $\eta = t_L/t_s$, t_L is the thickness of the layer, t_s is the thickness of the substrate; and $\chi = Y_L/Y_s$, Y_L is the Young's modulus of the layer, Y_s is the Young's modulus of the substrate.[3]

Simply *S* can be expressed as:

$$S = D/2R \tag{8}$$

Where *D* is the thickness of the substrate, *R* is the radius of curvature. Meanwhile, *L* is also related to strain which can be directly read from the screw micrometer.

Supplementary tables and figures

Supplementary Table 1. Surface conditions for different substrates

Substrate	Substrate Roughness	Temperatures (℃) for ALD Al_2O_3	Al_2O_3 Roughness
Si	0.23 nm	200	0.35 nm
ITO-coated PET	24.38 nm	70	32.59 nm

Supplementary Table 2. DFT calculations.

Orbitals	HOMO (eV)	LUMO (eV)	Gap (eV)
AZO-BTBT-8-*trans*	-5.676	-2.605	3.071
AZO-BTBT-8-*cis*	-5.668	-2.397	3.271

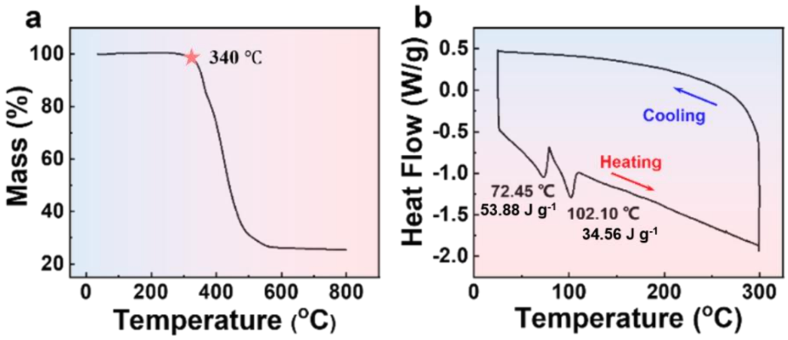

Supplementary Figure 2 | ** Thermodynamic Characterizations. TGA (a**) and DSC (**b**) plots for AZO-BTBT-8. Temperatures and enthalpies are listed around the peaks.

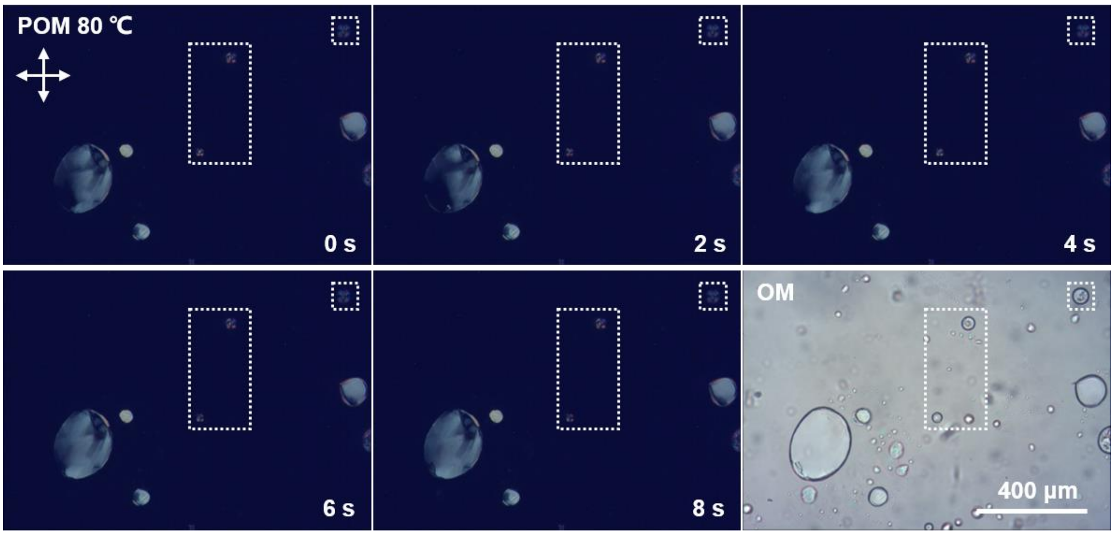

Supplementary Figure 3│The time-lapsed liquid crystal incubated at 80 ℃. The Maltese crosses are indicated by the dashed white rectangles. OM: optical microscopy; POM: polarized optical microscopy.

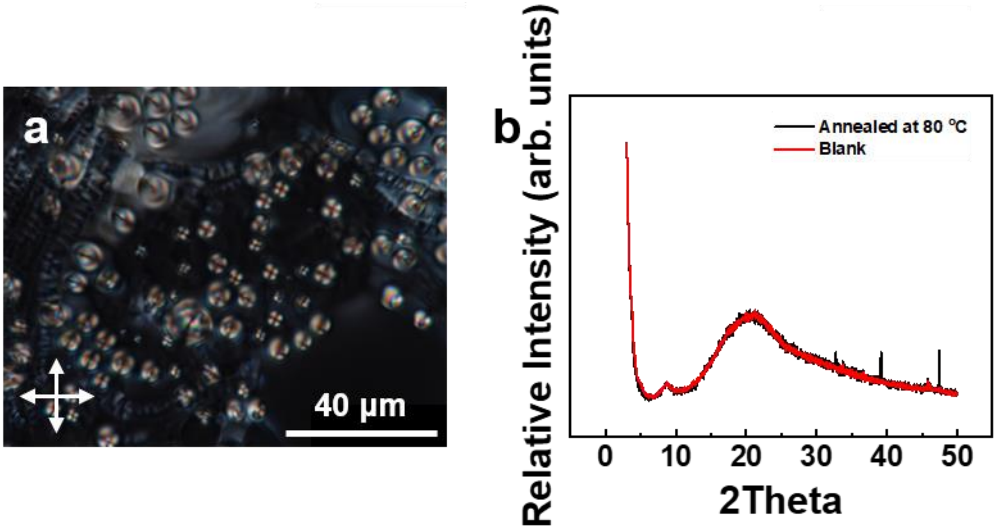

Supplementary Figure 4│liquid crystal tests. **a**, The Maltese crosses incubated at 80 ℃. **b**, XRD pattern for the mesophase incubated at 80 ℃.

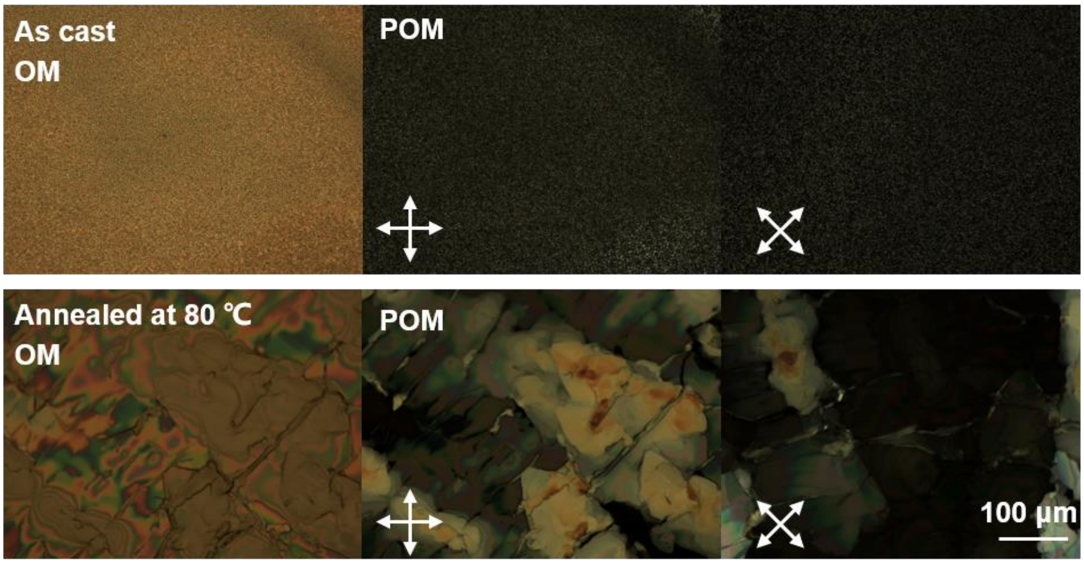

Supplementary Figure 5 │OM and POM images of the as-cast and annealed film samples. The right column shows the POM images of film samples rotated by 45 degrees.

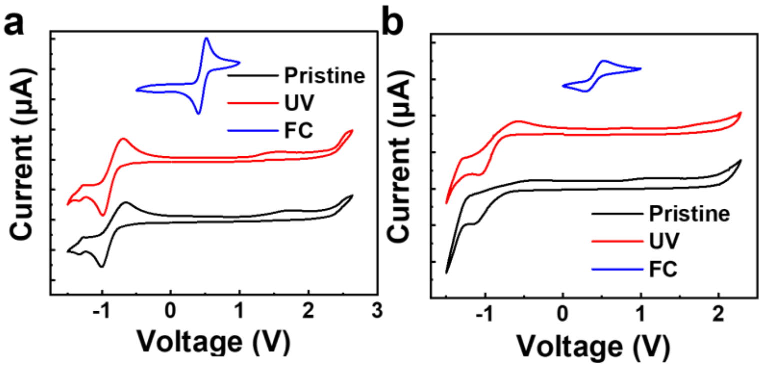

Supplementary Figure 6│ CV plots for the AZO-BTBT-8 solution (**a**) and the film sample (**b**).

Supplementary Table 3. Electrochemical and optical properties of AZO-BTBT-8[a]

	Solution				Solid Film			
	HOMO (eV)	LUMO (eV)	λ_{onset} (nm)	ΔE_g (eV)	HOMO (eV)	LUMO (eV)	λ_{onset} (nm)	ΔE_g (eV)
Pristine/trans	-5.837	-2.837	413.2	3.00	-5.492	-3.152	530.8	2.34
UV/Cis	-5.639	-2.539	388.7	3.10	-5.363	-2.993	523.2	2.37

[a]Abbreviation: HOMO = highest occupied molecular orbital derived by the equation: $\mathrm{HOMO} = -(E_{\mathrm{onset-ox}} + 4.8 - E_{\mathrm{ferrocene}})$ eV, λ_{onset} = onset absorption wavelength in UV-Vis spectra, ΔE_g = energy band gap determined from λ_{onset}, LUMO = lowest unoccupied molecular orbital = E_g + HOMO. $E_{\mathrm{onset\text{-}ox}}$ = onset oxidation potential measured by cyclic voltammetry in Supplementary Fig. 6.

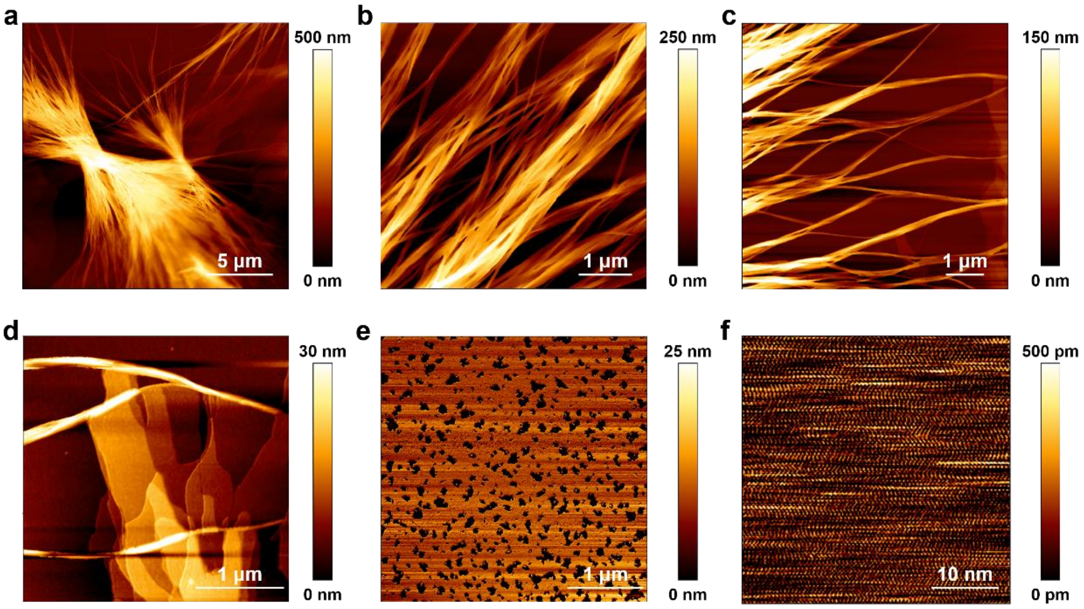

Supplementary Figure 7 | AFM images for different morphologies. **a-c,** wires; **d,** junction between wires and films; **e,** films. **f,** STM image of a large-scale assembly of AZO-BTBT-8 from octylbenzene on an HOPG surface. The tunneling current is 300 pA with a bias of -700 mV.

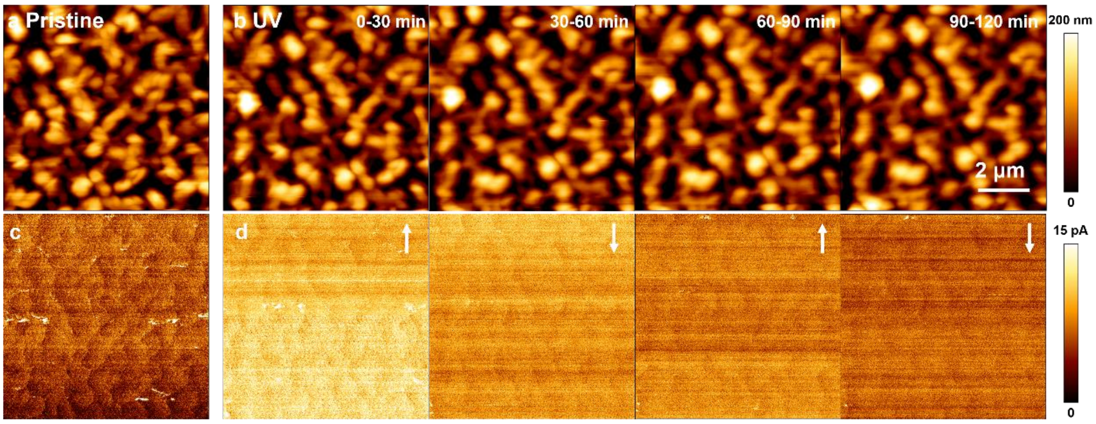

Supplementary Figure 8 | *In-situ* conductive AFM. **a** and **c** are morphology AFM and conductive AFM images for as-cast AZO-BTBT-8 film as comparison. **b** and **d** are *in-situ* morphology AFM and conductive AFM scanning decay images after UV radiation for 2 h. The white arrows indicate scanning directions.

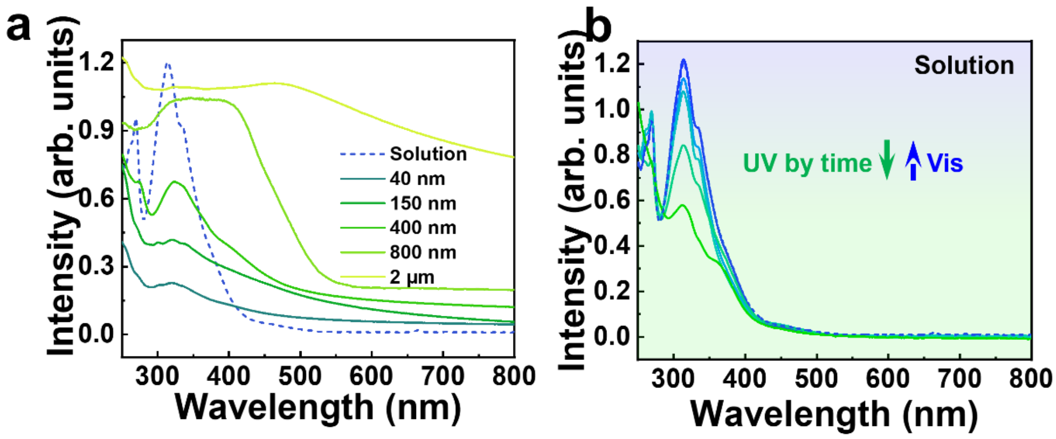

Supplementary Figure 9 | UV-Vis tests. **a**, UV-Vis comparison of solution samples and film solid samples with different thickness on quartz. **b**, Time-dependent UV-Vis spectra of a solution under ordinal UV and Vis irradiation. Green arrows represent the variation tendency of UV irradiation, and dashed blue arrows represent the variation tendency of visible light irradiation.

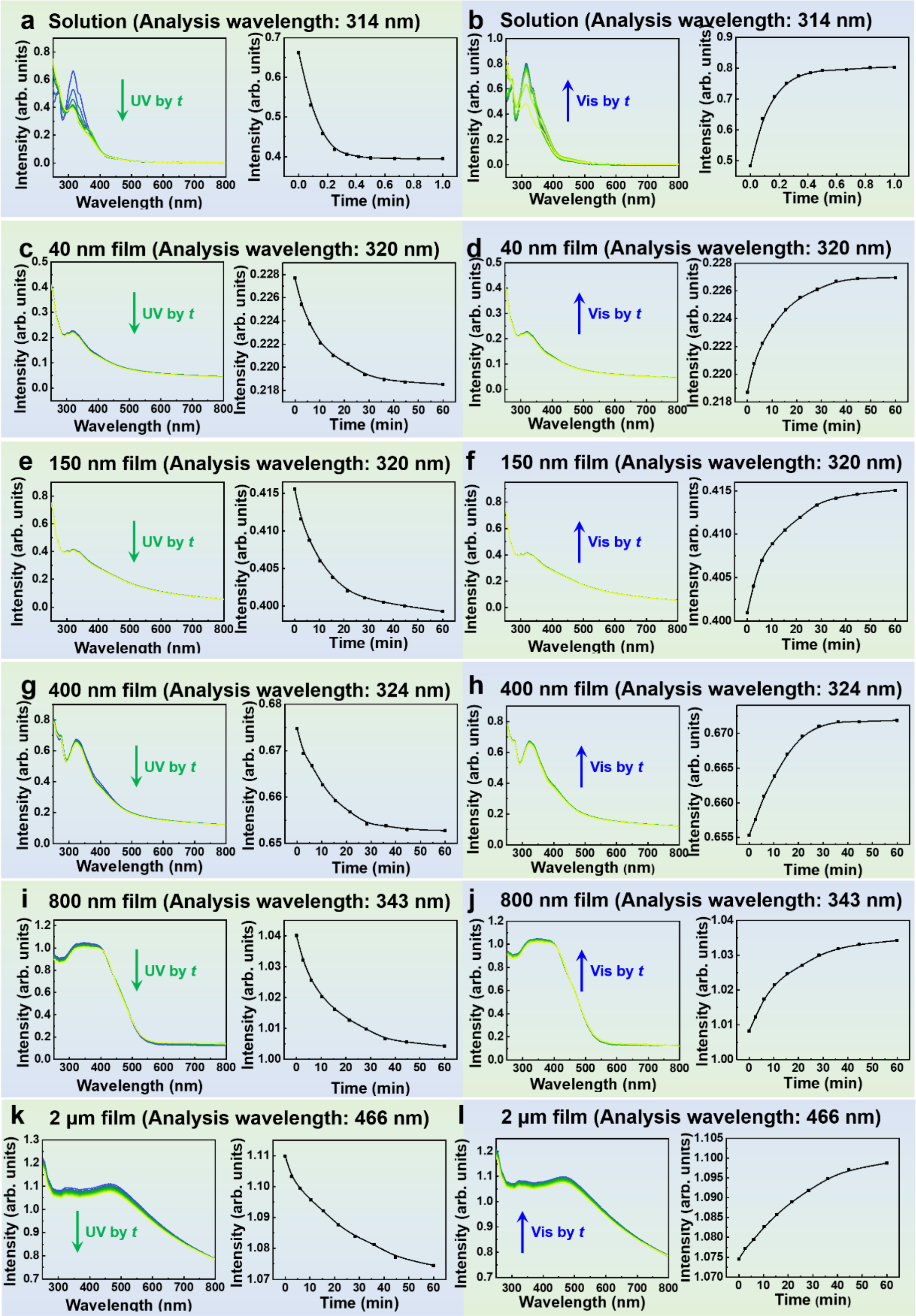

Supplementary Figure 10 | UV-Vis absorption spectroscopic studies. The gradual transitions of UV-Vis absorption spectra under UV (**a, c, e, g, i** and **k**) and visible light (**b, d, f, h, j** and **l**) irradiation. The left plots are the UV-Vis absorption spectra, and the right plots are the intensity extraction curves at the analysis wavelength from the left UV-Vis absorption spectra.

Supplementary Table 4. Fitting values for UV-Vis absorption spectroscopic studies in Supplementary Fig. 10.

	UV			Vis		
	k_{UV} (min^{-1})	$t_{1/2}$ (min)	Percent conversion (%)	k_{Vis} (min^{-1})	$t_{1/2}$ (min)	Percent conversion (%)
0 (Solution)	8.63231	0.0803	40.42	7.53623	0.0920	39.85
40 nm	0.08712	7.96	4.03	0.08248	8.40	3.63
150 nm	0.08691	7.98	3.92	0.07700	9.00	3.40
400 nm	0.07618	9.10	3.26	0.07504	9.24	2.46
800 nm	0.07228	9.59	3.25	0.06349	10.93	2.37
2 um	0.03818	18.15	3.23	0.03343	20.73	2.24

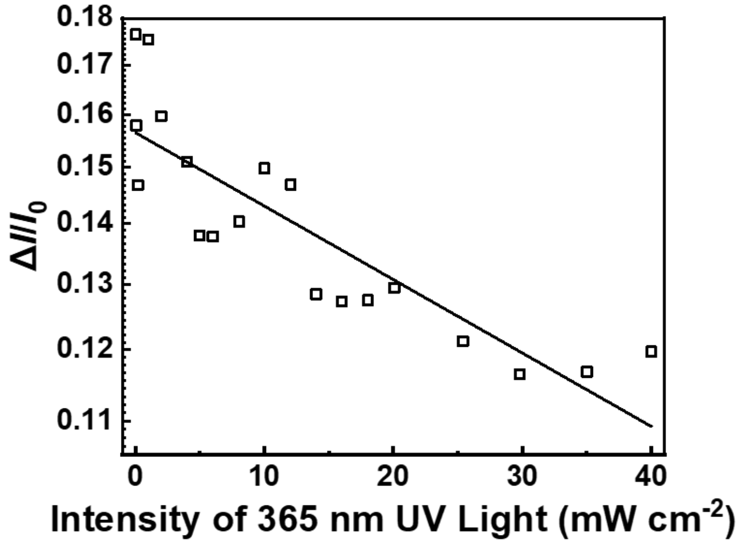

Supplementary Figure 11 | $\Delta I/I_0$ as a function of the UV intensity at 365 nm. ΔI, UV intensity absorbed by the film; I_0, the incident UV intensity.

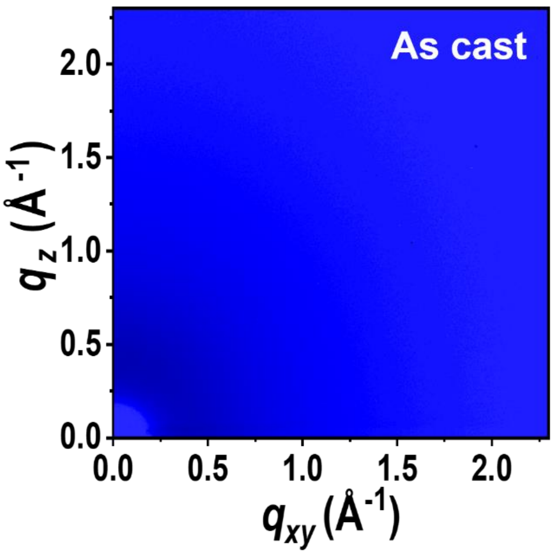

Supplementary Figure 12 | GIXD diffraction pattern for the as-casted film samples.

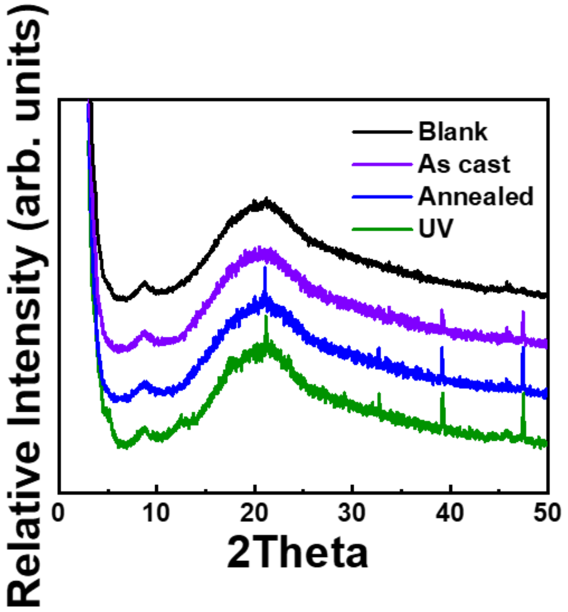

Supplementary Figure 13 | XRD patterns of the film samples.

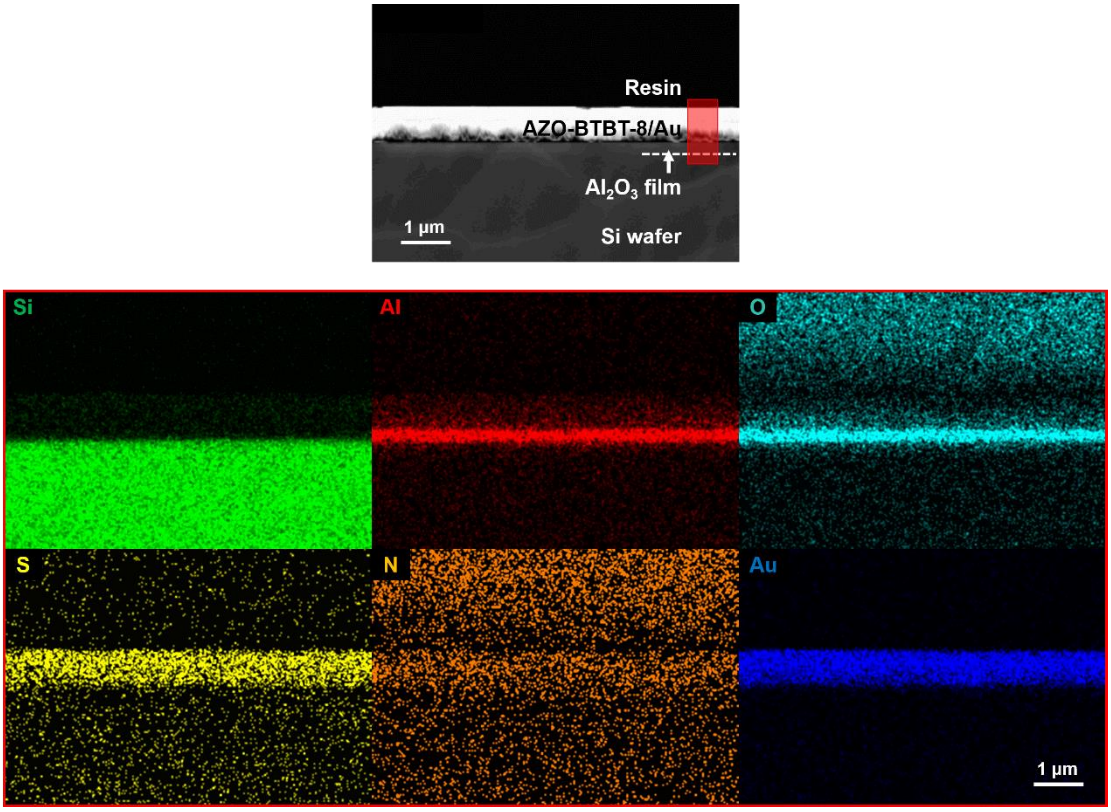

Supplementary Figure 14 | Cross-sectional SEM image of the OFET device. The sample was first embedded in resin, then prepared with a focused ion beam and imaged by SEM (200 kV). Below are analyses of the elemental compositions including silicon (green), aluminum (red), oxygen (lake blue), sulfur (yellow), nitrogen (orange) and gold (dark blue).

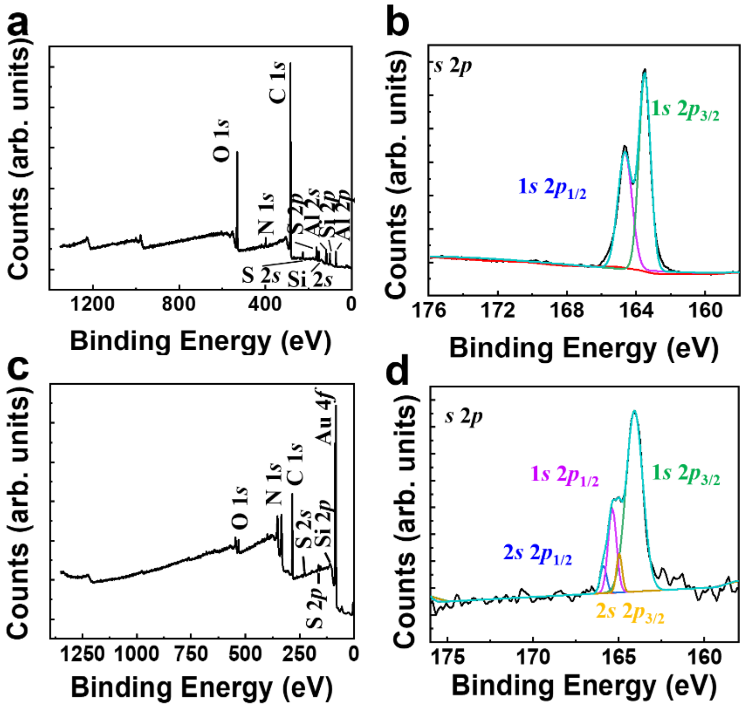

Supplementary Figure 15 │XPS surveys for AZO-BTBT-8 film before (**a-b**) and after Au electrode deposition (**c-d**). **b** and **d** are high resolution XPS for S 2*p*.

Supplementary Table 5. Fitting values for transition studies on temperatures.

		k (min^{-1})	$t_{1/2}$ (min)
Conductive AFM	RT	0.01587	43.68
Current measurement	RT	0.03073	22.56
	RT-UV@80 °C	0.01887	36.73
	30 °C	0.05528	12.54
	35 °C	0.07926	8.75
	40 °C	0.1115	6.22
	Vis	0.06523	10.63

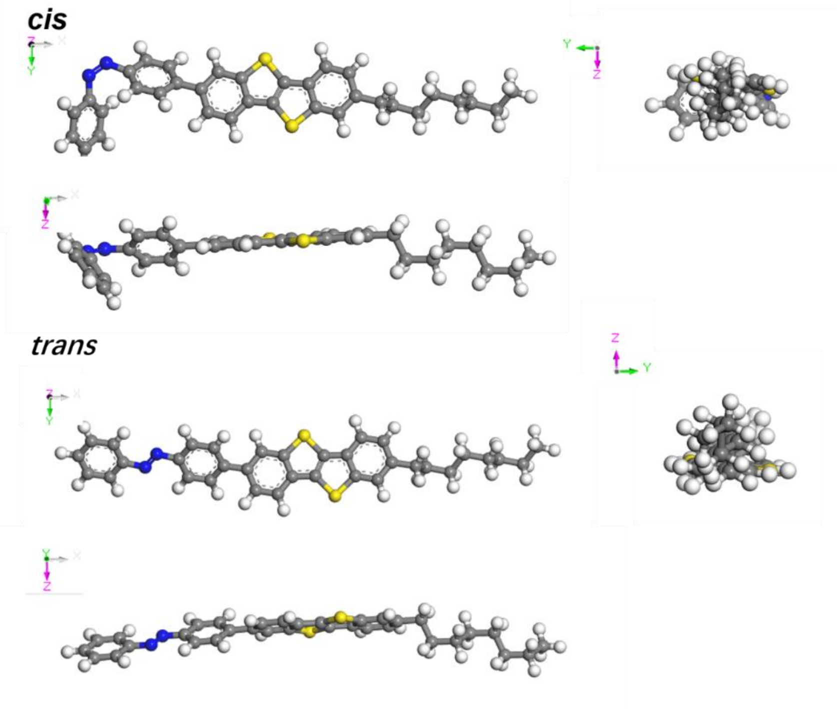

Supplementary Figure 16 | DFT molecular structures of AZO-BTBT-8 in both *cis* and *trans* conformations.

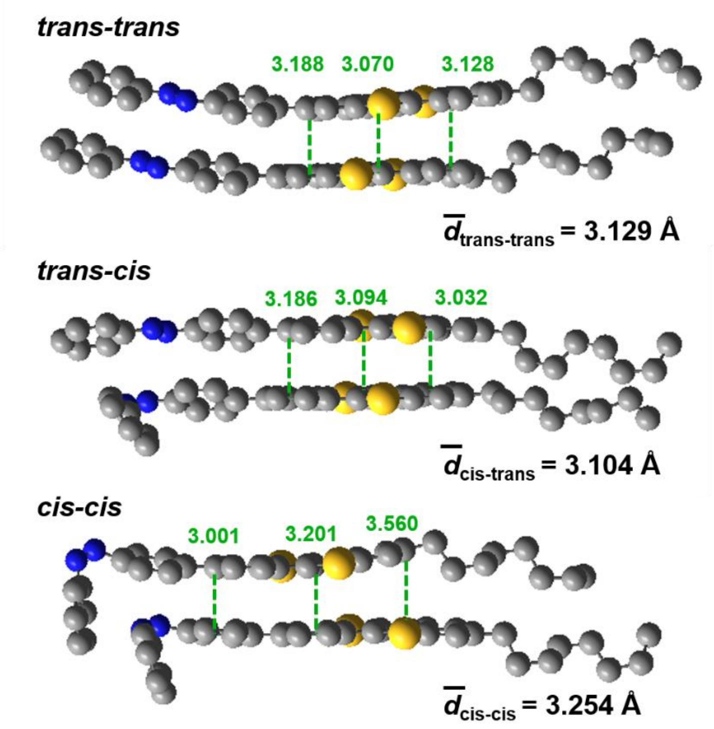

Supplementary Figure 17 | Distance measurements between conformations in DFT calculations. The average distances between conformations is *cis-cis* (3.254 Å) > *trans-trans* (3.129 Å) > *trans-cis* (3.104 Å)

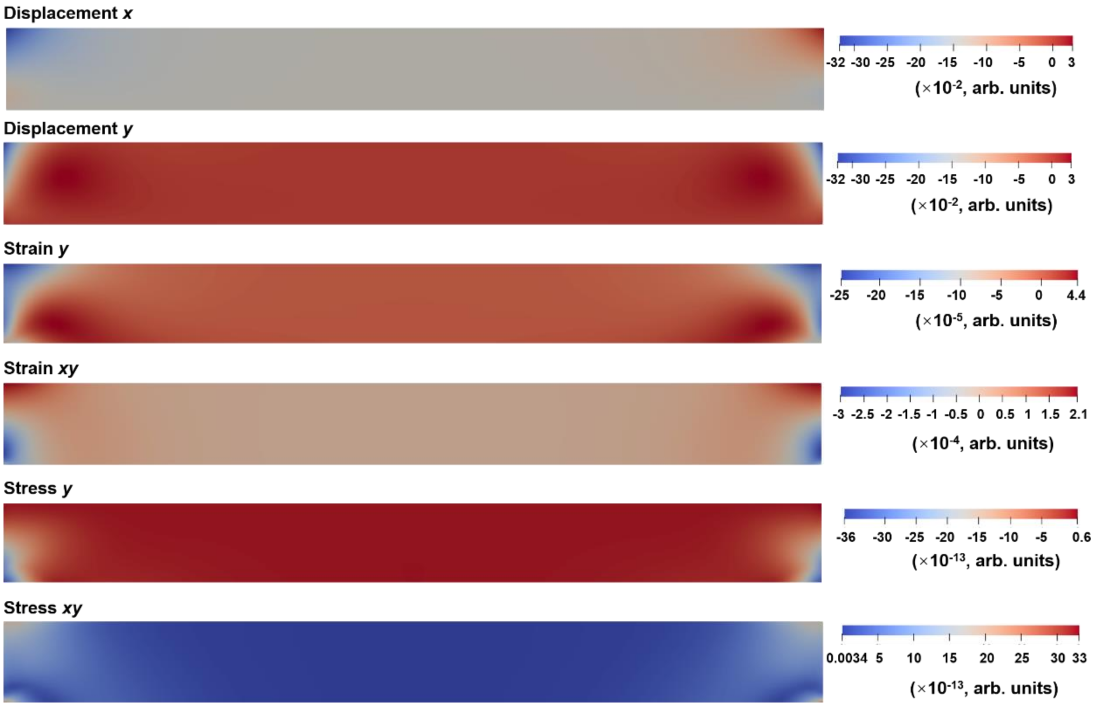

Supplementary Figure 18 | Schematic diagram of the displacement, stress and strain distributions of the film section from mechanical calculation

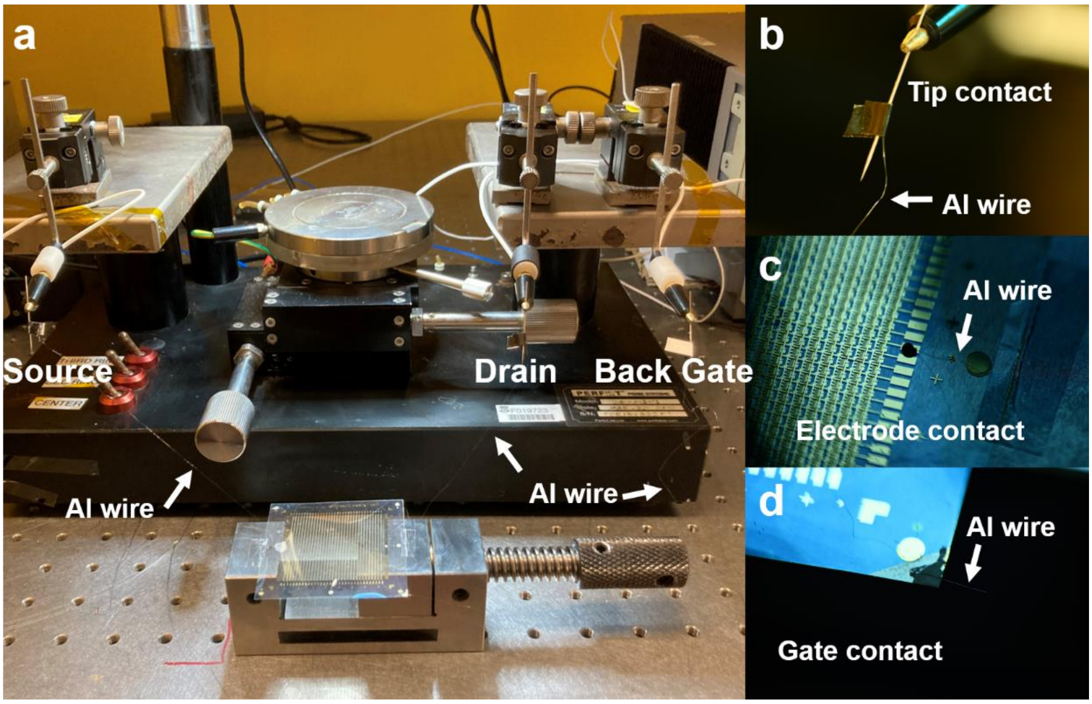

Supplementary Figure 19 | Setups for OFET tests. **a,** Setups of device characterizations. Wire bonding images for tip contact (**b**), electrode contact (**c**) and gate contact (**d**).

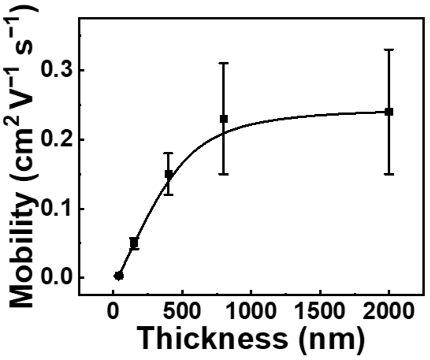

Supplementary Figure 20 | Mobility curve of OFET device on silicon substrate with thickness dependence.

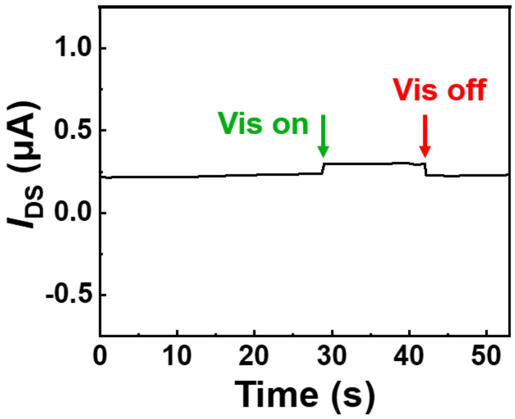

Supplementary Figure 21 | Time trace of I_{DS} for the device under Vis irradiation. V_{DS} = -25 V, V_G = -25 V. Vis = visible (λ ≥ 420 nm).

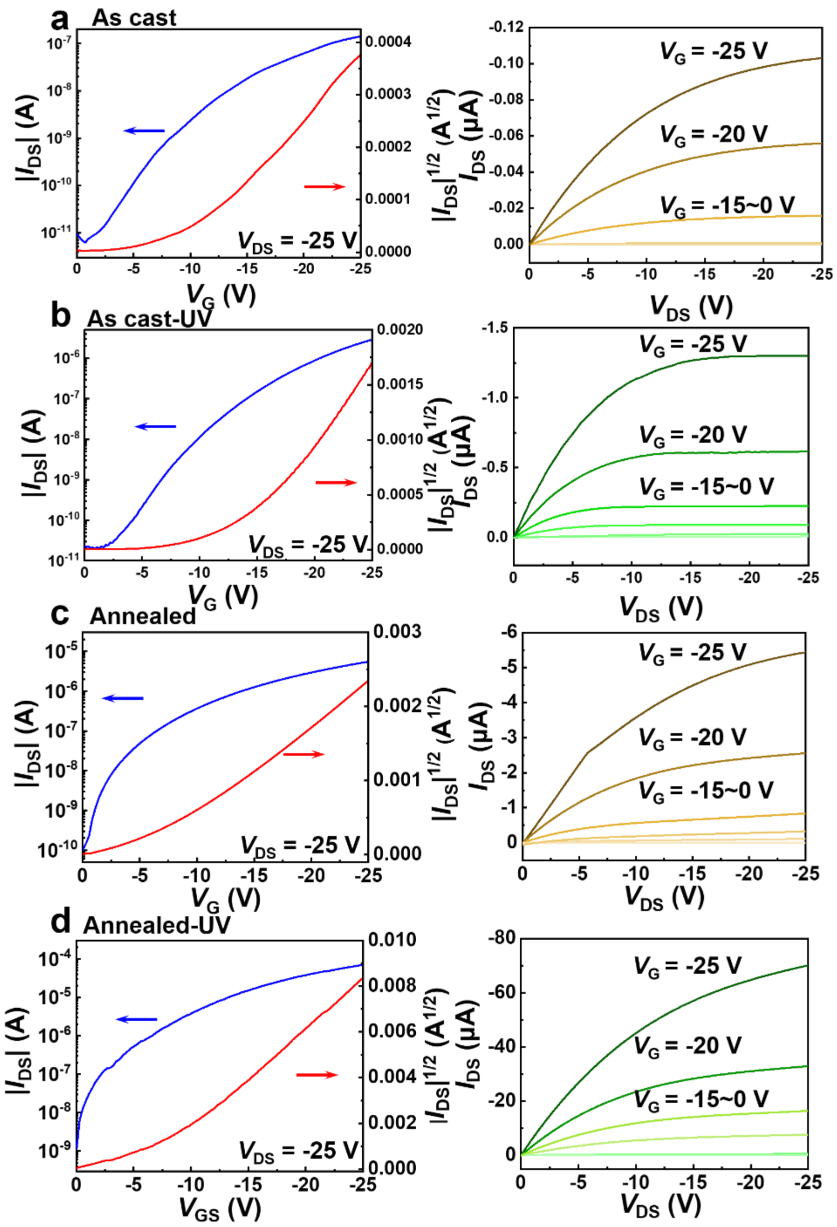

Supplementary Figure 22 | OFET properties on silicon substrates. The transfer (left) and output curves (right) of AZO-BTBT-8 for (a) as-casted films, (b) as-casted films under UV irradiation, (c) annealed films and (d) annealed films under UV irradiation. The average on/off ratio is approximately 10^5. L = 30 μm and W = 130 μm.

Supplementary Table 6. Mobilities of the OFETs on silicon substrate in Supplementary Fig. 22

OFET devices	Mobility (cm^2 V^{-1} s^{-1})	
	Before UV irradiation	After UV irradiation
Without thermal annealing	0.0071± 0.001	0.19± 0.07
With thermal annealing	0.23 ± 0.08	3.80 ± 0.09

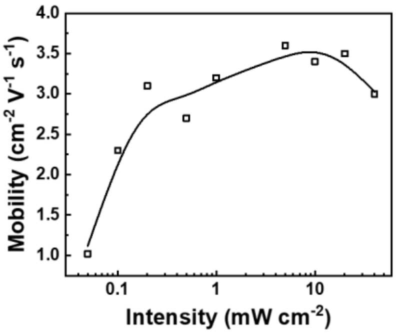

Supplementary Figure 23 | Carrier mobility on silicon substrate as a function of the UV intensity at 365 nm for 20 min.

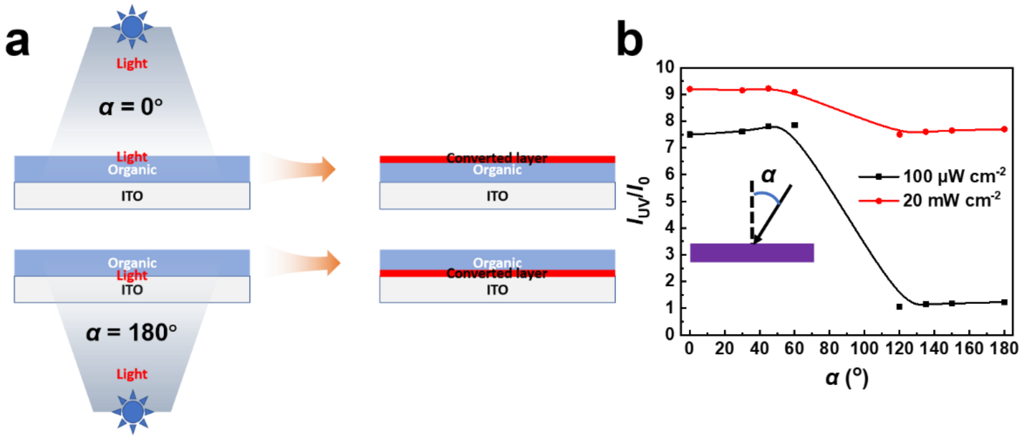

Supplementary Figure 24 | The angular-dependent photoresponse experiments. **a,** Schematic image for UV irradiation with different incident angles; **b,** The incident angle dependent response plot. I_0 and I_{UV} are current intensities before and after UV irradiation.

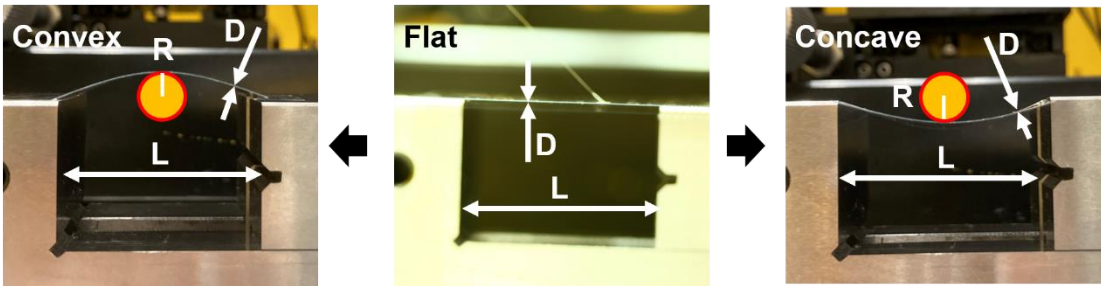

Supplementary Figure 25 | Optical images for convex and concave bending device.

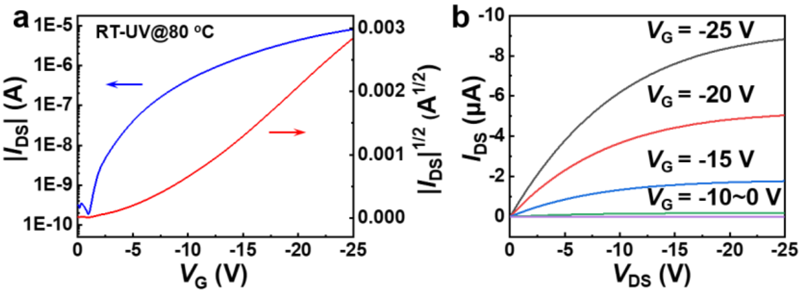

Supplementary Figure 26 | FET properties. **a** and **b** are the transfer and output curves of AZO-BTBT-8 at room temperature, after UV irradiation at 80 °C for 30 min on PET. The mobility are 0.367 ± 0.20 $cm^2\ V^{-1}\ s^{-1}$. The on/off ratio is approximately 10^5. L = 30 μm and W = 130 μm.

Supplementary References

1. Harvey, J.H., Butler, B.K. & Trauner, D. Functionalized azobenzenes through cross-coupling with organotrifluoroborates. *Tetrahedron Letter* **48**, 1661-1664 (2007).

2. Kpaata, B., Kozmík, V. & Svoboda, J.í.J.C.o.C.C.C. Reactivity of [1]Benzothieno[3,2-b][1]benzothiophene - Electrophilic and Metallation Reactions. *Collection of Czechoslovak Chemical Communications* **67**, 645-664 (2002).

3. Gleskova, H., Wagner, S., Soboyejo, W. & Suo, Z. Electrical response of amorphous silicon thin-film transistors under mechanical strain. *Journal of Applied Physics* **92**, 6224-6229 (2002).
